# Supplementary material for: WISP1 Is Involved in the Pathogenesis of Kashin-Beck Disease via the Autophagy Pathway
Source: Int J Mol Sci. 2023 Nov 7;24(22):16037. doi: 10.3390/ijms242216037 (PMC10671535; doi:10.3390/ijms242216037)
Supplement: Supplementary file 1 [file ijms-24-16037-s001.zip › Supplementary file legends.pdf]

Supplementary files

Supplementary Table S1. Basic characteristics of study samples

Supplementary Figure S1. The result of MTT assay

Five different T-2 concentrations (1, 2, 5, 8, 10ng/ml) of T-2 toxin and three different times (12h, 24h, 72h) were set to intervene in C28/I2 cell line. The median inhibitory concentrations (IC50) of T-2 toxin were 25.14 ng/ml, 12.66ng/ml, and 4.96 ng/ml for 12h, 24h and 72h, respectively, and 24h (P=0.028) and 72h (P=0.001) showed significant differences.

Supplementary Table S2. The primer sequences of study genes in qPCR
