# Supplementary material for: WISP1 Is Involved in the Pathogenesis of Kashin-Beck Disease via the Autophagy Pathway
Source: Int J Mol Sci. 2023 Nov 7;24(22):16037. doi: 10.3390/ijms242216037 (PMC10671535; doi:10.3390/ijms242216037)
Supplement: Supplementary file 1 [file ijms-24-16037-s001.zip › Supplementary TAble S1.pdf]

Table S1. Basic characteristics of study samples

Group	Samples	Age(Mean±SD)	Male/Female	Normal Distribution		T-test
				Y/N	*P* value	
1	KBD	53.0±3.0	2/1	/	/	0.921
	Control	54.0±16.09	1/2	/	/	
2	KBD	57.4±7.95	1/4	Y	0.200	0.245
	Control	66.2±13.55	1/4	Y	0.182	
3	KBD	44.0	0/1	/	/	/

Note: Group 1 was for tissue staining and IHC, group 2 was for qPCR and WB, and group 3 was for TEM.
