# Supplementary material for: WISP1 Is Involved in the Pathogenesis of Kashin-Beck Disease via the Autophagy Pathway
Source: Int J Mol Sci. 2023 Nov 7;24(22):16037. doi: 10.3390/ijms242216037 (PMC10671535; doi:10.3390/ijms242216037)
Supplement: Supplementary file 1 [file ijms-24-16037-s001.zip › Supplementary Table S2.pdf]

Table S2. The primer sequences of study genes in qPCR

Gene Name		Sequence(5′-3′)
ATG4A	F	CAGATGAGCTGGTATGGATCTTAGG
	R	TGGGCTGTTAGCAGTTTCAGG
ATG4B	F	AGCCTGGCTTACCAAGGGCTAC
	R	GTCCTGTGCACGCCATCTTC
ATG4C	F	GACAGTTTGATTTACATGGATCCTC
	R	GCTCGTTTGAAGTCCTGAACA
WISP1	F	GGGAAGAAGTGTCTGGCTGTG
	R	TGGGTTGATAGGAGCGTGTG
BECN1	F	CCAGATGCGTTATGCCCAGAC
	R	CATTCCATTCCACGGGAACAC
MAP1LC3A	F	TTCCTGAACTGAGCTGCCTCTAC
	R	ACCCAGAGGGACAACCCTAAC
MAP1LC3B	F	AGTTGGCACAAACGCAGGGTA
	R	TTAGGAGTCAGGGACCTTCAGCA
COL2A1	F	AGACTGGCGAGACTTGCGTCTA
	R	ATCTGGACGTTGGCAGTGTTG
SOX9	F	GGAGATGAAATCTGTTCTGGGAATG
	R	TTGAAGGTTAACTGCTGGTGTTCTG
ACAN	F	ACGAAGACGGCTTCCACCAG
	R	TCGGATGCCATACGTCCTCA
GPADH	F	GCACCGTCAAGGCTGAGAAC
	R	TGGTGAAGACGCCAGTGGA
