# Supplementary figures and images for: WISP1 Is Involved in the Pathogenesis of Kashin-Beck Disease via the Autophagy Pathway
Source: Int J Mol Sci. 2023 Nov 7;24(22):16037. doi: 10.3390/ijms242216037 (PMC10671535; doi:10.3390/ijms242216037)

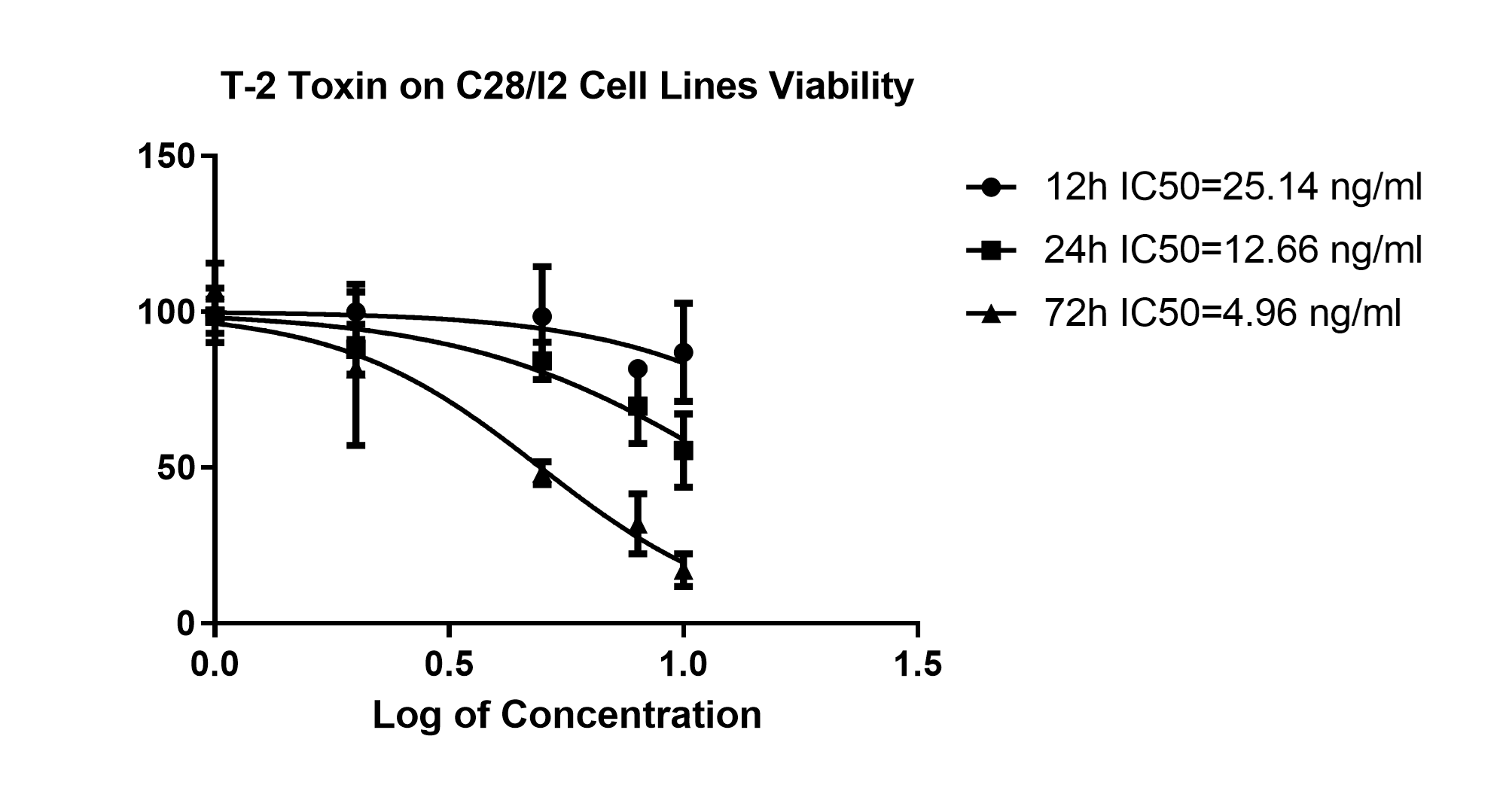

Supplement: Supplementary file 1 [file ijms-24-16037-s001.zip › Supplementary Figure S1.tif]
